# Association between Serum Lipid Parameters and Cognitive Performance in Older Adults

**DOI:** 10.3390/jcm10225405

**Published:** 2021-11-19

**Authors:** Jaeho Lee, Sohyae Lee, Jin-young Min, Kyoung-bok Min

**Affiliations:** 1 Department of Preventive Medicine, College of Medicine, Seoul National University, Seoul 03080, Korea; jhtyu21@naver.com (J.L.); leesohyae@gmail.com (S.L.); 2 Integrated Major in Innovative Medical Science, Seoul National University Graduate School, Seoul 03080, Korea; 3 Veterans Medical Research Institute, Veterans Health Service Medical Center, Seoul 05368, Korea; 4 Institute of Health Policy and Management, Seoul National University Medical Research Center, Seoul 03080, Korea

**Keywords:** cognitive function, lipid, high-density lipoprotein, cognitive performance, aging

## Abstract

(1) Background: Previous studies have suggested the association between lipid profiles and cognitive function in older adults. However, they generated inconsistent results. We aim to determine the relationship between lipid profiles and cognitive performance in older adults. (2) Methods: We used the 2011–2014 National Health and Nutrition Examination Survey. This study included 2215 participants who were aged more than 60 years old and free of coronary heart disease or stroke. Lipid profiles included total cholesterol (TC), low density lipid cholesterol (LDL), high density lipid cholesterol (HDL), and triglyceride (TG). Cognitive function was assessed using the digit symbol substitution test (DSST). (3) Results: Positive correlations of DSST were observed with TC (r = 0.111; *p* < 0.0001), HDL (r = 0.127; *p* < 0.0001), and LDL (r = 0.107; *p* = 0.0005). However, there was no significant relationship between TG and DSST. A one-unit increase in HDL was associated with an increase in DSST score (beta coefficient: 0.036; *p* = 0.018); but the association was not significant for LDL, TG, and TC. In the categorical analysis, the high HDL group had a higher DSST score than the low HDL group (beta = 3.113; *p* < 0.0001) and the low TG group was more likely to show a lower DSST score than the high TC group (beta = −1.837; *p* = 0.0461). However, LDL and TC showed no statistically significant associations. Moreover, HDL was only associated with a 0.701 times increased risk of cognitive impairment (95% CI = 0.523–0.938) in the logistic regression analysis. (4) Conclusions: Higher blood concentrations of HDL levels were positively associated with DSST scores in older adults. We suggest that the high levels of HDL may be a protective factor against cognitive impairment.

## 1. Introduction

Cognitive decline is an important public health issue among older adults. Approximately 20%−30% of Americans and 8% of Chinese in the elderly population have health issues related to cognitive impairment [1]. Abnormal cognitive functioning impedes the quality of life and affects the disease burden for caregivers as well as patients [2]. It is more likely to further develop into worse impairments, such as Alzheimer’s disease or dementia, and increase the risk of morbidity and mortality [3,4,5]. Although there is no certain treatment to prevent cognitive impairment, the risk of cognitive decline is often ignored until patients are diagnosed with final progression to dementia [5].

Several risk factors are involved in the development of cognitive decline. These include health behaviors (i.e., cigarette smoking and dietary intake) and comorbidities (i.e., hypertension and diabetes mellitus) [6]. For example, a Chinese cohort study indicated that cognitive impairment has a significant association with past smokers (relative risk (RR) = 0.73, 95% confidence interval (CI) = 2.22–2.81) and current smokers (RR = 2.33, 95% CI = 1.37–5.82) compared to non-smokers who have never smoked in their entire life [7]. A community-based cohort study found that the presence of diabetes was negatively associated with the mean change scores for the digit symbol subtest (DSST) and first-letter word fluency (WF). Hypertension was also significantly associated with higher negative mean change scores in WF [8]. A study of elderly individuals in Madrid reported that the ingestion of a satisfactory global diet and balanced nutrients was associated with better cognitive function [9].

Lipid profiles are another important risk factor for cognitive impairment [10,11]. Plasma lipid levels were higher in individuals with mild cognitive impairment than in those with normal cognition; herein, the occurrence was associated with elevated plasma high-density lipoprotein cholesterol (HDL) and triglycerides (TG) [10]. Lower levels of total serum cholesterol were associated with poor performance in terms of verbal fluency, abstract reasoning, attention/concentration, and a composite score of multiple variables [12]. A cross-sectional study in China showed that subjects with high levels of TC and low-density lipoprotein cholesterol (LDL), but not HDL and TG, were at risk for cognitive impairment [13]. Another prospective cohort study in the United States indicated that LDL levels were associated with good performance in cognitive memory [14]. Although the above-mentioned studies have suggested that multiple serum lipid parameters are a risk factor for cognitive impairment, the results are inconsistent. Thus, further studies are needed to define the association between serum lipid parameters and cognitive impairment.

In this study, we conducted a cross-sectional study using the National Health and Nutrition Examination Survey (NHANES), a representative sample of the United States. Our study aimed to determine whether lipid profiles (i.e., TC, LDL, HDL, and TG) were associated with cognitive performance in older adults. Moreover, this study aimed to consider whether lipid profiles could be the predictors for cognitive function. The DSST, which is known to be one of the most sensitive tests of cognitive dysfunction, was used to assess cognitive performance [15].

## 2. Materials and Methods

### 2.1. Study Population

We used data from the NHANES 2011–2014. The NHANES conducted by the Centers for Disease Control and Prevention is a nationally representative survey of the non-institutionalized civilian population in the United States.

From the 2011–2014 NHANES data, 3632 participants aged >60 years were initially included. We then selected 2606 patients who had data on blood lipid examination and who completed the DSST test. We further excluded 391 subjects who had experienced coronary heart disease or stroke and who had no variables of interest. Finally, we included a total of 2215 people as study participants. The study protocols of the NHANES were approved by the National Center for Health Statistics Institutional Review Board. Oral, and written consent was obtained from all the participants before the survey.

### 2.2. Measurement of Serum Lipids Profiles

Serum samples were collected and analyzed using different methods depending on the characteristics of the serum lipids. TC and TG levels were measured enzymatically [16]. HDL cholesterol was measured in serum after rendering apolipoprotein-B-containing lipoproteins non-reactive with a blocking agent [17]. Cholesterol was calculated from the measured values of TC, TG, and HDL cholesterol using the Friedewald formula [17]. The cut-off values for distinguishing normal versus abnormal HDL, LDL, TG, and TC levels were 40 mg/dL, 130 mg/dL, 150 mg/dL, and 200 mg/dL, respectively, based on criteria of the UK consensus group and recent Europeans guidelines [18,19].

### 2.3. Measurement of DSST

Although relying on cognitive assessment cannot fully replace a diagnosis based on a clinical examination, cognitive assessment is helpful to understand the association of cognitive functioning with regards to risk factors of measuring cognitive impairment or further severe cognitive symptoms such as dementia.

The DSST, which is a performance module from the Wechsler Adult Intelligence Scale, assesses sustained attention, processing speed, and working memory [14]. The test form had a key at the top, containing nine numbers paired with symbols. Participants were given 2 min to copy the corresponding symbols in the 130 boxes that adjoined the numbers. The interviewer showed the participants how to complete the task before the initial assessment and then requested them to fill in the practice boxes. Participants who were unable to finish the practice were excluded from the study. The score calculates the total number of correct matches. The values for the score ranged from 0 to 100 [20]. Participants with DSST scores less than 33 were classified as cognitively impaired, given that this range of values belongs to the lowest quartile of the distribution.

### 2.4. Variables of Interest

Variables of interest included age (>60 years), sex (male or female), race/ethnicity (non-Hispanic white, non-Hispanic black, Hispanic, or other), annual household income (<$20,000 or ≥$20,000), and education (non-high school graduate, high school graduate, or college graduate or over). Furthermore, smoking status was classified as current smoker, former smoker, or never smoker. Exercise habits were classified as regular or non-exercise-based. Body mass index (BMI) was calculated by dividing the individual’s weight by his or her height squared and categorized into the following four groups: underweight (<18.5 kg/m^2^), normal weight (18.5–22.9 kg/m^2^), overweight (23–24.9 kg/m^2^), and obese (≥25 kg/m^2^).

Medical condition variables were divided into binary variables (Yes/No). The presence of depressive feelings was evaluated based on the questionnaire responses: participants who experienced feeling low, depressed, or hopeless over the last 2 weeks. Diabetes was classified according to the two-hour glucose (OGTT) levels: Yes (≥200 mg/dL) and No (<200 mg/dL). The presence of chest pain was determined based on the questionnaire responses: participants who experienced any pain or discomfort in chest. Hypertension was determined by high diastolic blood pressure (≥90 mm Hg) or systolic blood pressure (≥140 mm Hg). The use of statin was determined by whether any of the medications including simvastatin, lovastatin, atorvastatin, rosuvastatin, pravastatin, and pitavastatin, was taken in the 30 days prior to taking the questionnaire.

### 2.5. Statistical Analysis

The mean values and standard deviations of lipid profiles and cognitive test scores were computed. The correlation coefficients of the lipid profiles with cognitive test scores and among the lipid profiles were also calculated. To evaluate the association between lipid profiles and the DSST, linear regression, logistic regression, and spline regression analyses were conducted. The regression model provided the estimated beta values of the DSST in terms of TC, TG, HDL, and LDL. The logistic model provided the odds ratio estimates and 95% Wald Confidence Limits. The model was adjusted for age, sex, ethnicity, monthly income, education, smoking status, BMI, exercise, depression levels, diabetes, chest pain, hypertension, and statin.

We used weighted estimates of the population parameters based on the NHANES analytic and reporting guidelines. Statistical analyses were performed using the PROC SURVEY, PROC LOGISTIC, and PROC GAM procedure in SAS 9.2 (SAS Institute, Cary, NC, USA) to account for the complex sampling scheme. All the tests were two-sided, and the level of statistical significance was set to α = 0.05.

## 3. Results

### 3.1. Characteristics of the Study Population

Table 1 summarizes the characteristics of the study participants. Of the 2606 participants, 391 who had experienced coronary disease and stroke were excluded from the study. The mean age was approximately 69 years, and 52.73% of all subjects were female individuals. There were relatively few Asians (8.26%); 47.22% of the subjects were white individuals. The proportion of subjects who had a total annual family income ≥$20,000 was 75.76%, and the rest of the population belonged to the <$20,000 annual family income category. More than half (53.18%) of the subjects had higher levels of education with a college degree. As far as health behavior was concerned, more than half (51.38%) were current smokers, and 12.33% of the subjects had no experience of smoking at all. With regard to the health condition, 59.73% of the subjects reported exercising regularly, although 73.05% of the subjects showed an abnormal weight, including obesity, and overweight. Concerning medical conditions, most participants showed no depressive feelings (77.16%), diabetes (95.44%), chest pain (79.10%), hypertension (64.75%), or experience of taking statin (62.21%).

### 3.2. Comparison of the Means (SD) of Lipid Profiles and Cognitive Test Score

Table 2 presents the mean (±standard deviation (SD)) of lipid profiles (HDL, LDL, TG, and TC) and cognitive test scores for the DSST. The mean values of HDL, LDL, TG, and TC were 55.11 mg/dL, 113.05 mg/dL, 120.89 mg/dL, and 194.61 mg/dL, respectively. The mean score of the cognitive test for the DSST was 47.27 (range: 0–105).

**Figure 1:**
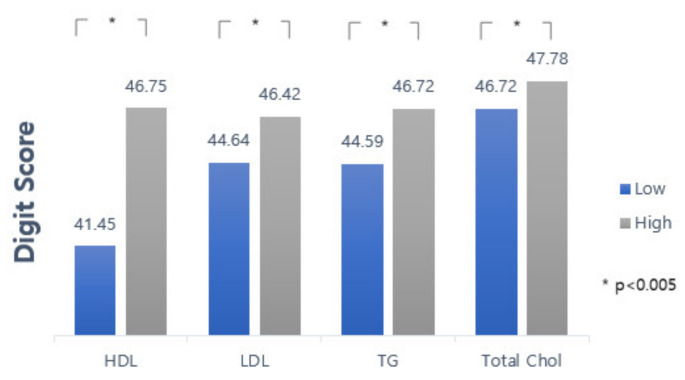
Comparison of the mean (±SD) cognitive test score (Digit Score) according to the abnormal baseline of different lipid profiles. Upper bar connecting to each side of the graph represents a significant difference using *p*-value.

Figure 1 categorical levels of all lipid profiles. Overall, the participants who had a high lipid profile presented high scores on the cognitive test.

**Figure 2:**
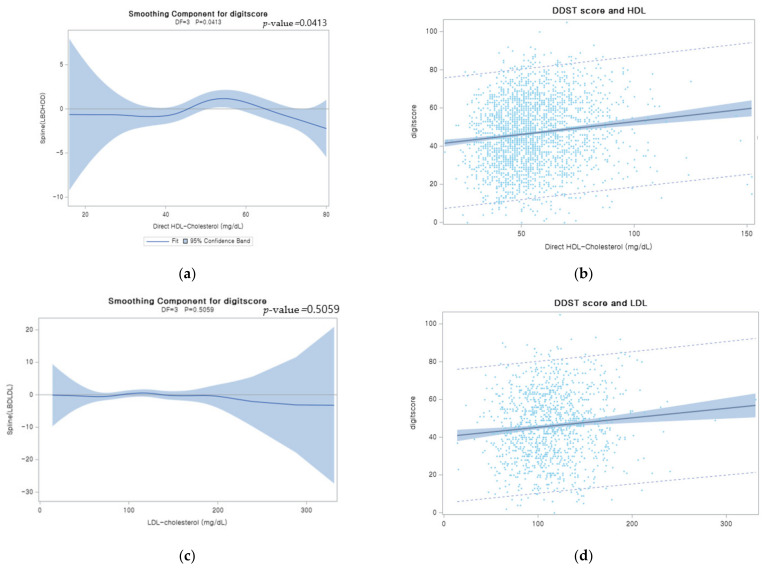

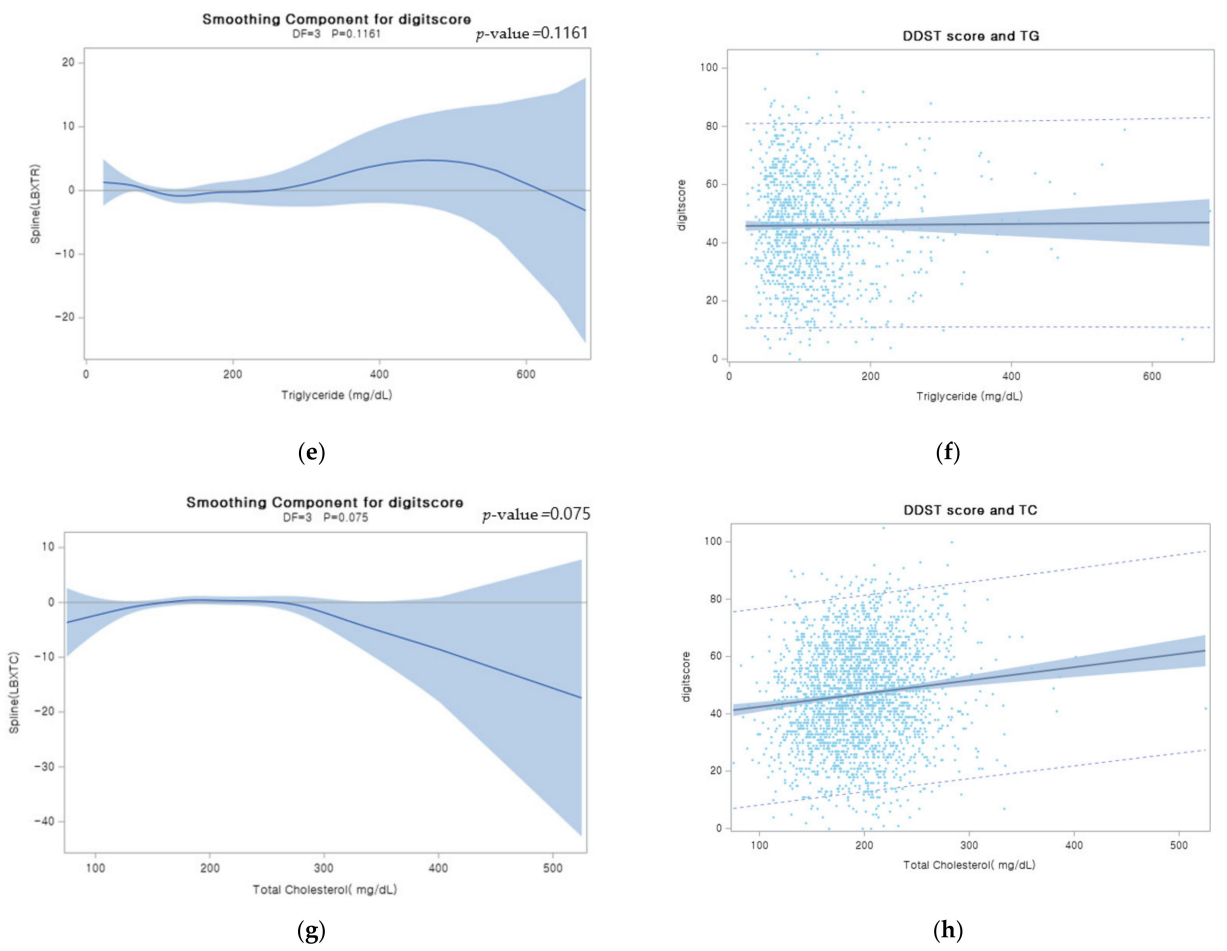
Plots of smoothing spline function of digit score (**a**) Plots of estimated smoothing spline function of digit score with 95% confidence band for Generalized Additive Model (GAM) when the independent variable was HDL. (**b**) Scatter plots of HDL when the independent variable was HDL. Smoothing spline estimated on the basis of the distribution of digit score and HDL. Light blue color band indicated 95% confidence interval range of the distribution. (**c**) Plots of estimated smoothing spline function of digit score with 95% confidence band for Generalized Additive Model (GAM) when the independent variable was LDL; (**d**) scatter plots of HDL when the independent variable was LDL. (**e**) Plots of estimated smoothing spline function of digit score with 95% confidence band for Generalized Additive Model (GAM) when the independent variable was TG. (**f**) Scatter plots of HDL when the independent variable was TG. (**g**) Plots of estimated smoothing spline function of digit score with 95% confidence band for Generalized Additive Model (GAM) when the independent variable was TC. (**h**) Scatter plots of HDL when the independent variable was TC.

Figure 2 shows a scatter plot and the estimated smoothing spline function of the digit score with a 95% confidence band for the generalized additive model (GAM) when the independent variable was lipid profiles. As visualized in Figure 2a,b, the estimated smoothing spline function of the digit score for the GAM only showed a significant relationship with HDL among lipid profiles (*p*-value: 0.0413). Our analysis revealed that those who have higher levels of HDL tended to show better performance on cognitive tests according to the scatter plot and the GAM figure.

### 3.3. Pearson Correlation Structure of Lipid Profiles-DSST and among Lipid Profiles

Table 3 shows the correlation coefficient of lipid profiles with cognitive test scores and among lipid profiles. All lipid profiles, except TG, showed a significant correlation. Specifically, the correlation between DSST scores and HDL showed a strong positive relationship (0.127) with a significant *p*-value (*p* < 0.05) compared to other lipid profiles. LDL (0.107) and TC (0.111) also showed a positive correlation coefficient with the DSST. TG levels showed no significant correlation. Overall, HDL showed a strong positive correlation coefficient relationship with cognitive test scores among the lipid profiles.

The correlation coefficient of all the pairs of lipid profiles showed a significant *p*-value. Particularly, LDL-TC presented a positive correlation coefficient (0.925) and HDL-TG showed a negative correlation coefficient (−0.419). Other correlation coefficients among lipid profiles presented a weak correlation.

### 3.4. Multiple Linear Regression and Logistic Regression Analysis of the Association between Lipid Profiles and DSST

Table 4 is divided into two parts according to the lipid profiles based on the continuous and categorical values. We analyzed multiple linear regression and logistic regression to examine the association between lipid profiles and DSST. The DSST score showed a positive association with an increase in one unit of HDL (beta coefficient: 0.036). However, other lipid profiles showed no significant relationship with the cognitive test scores. For categorical values, we adopted the same scale as the baseline for each lipid profile (i.e., 40 mg/dL, 130 mg/dL, 150 mg/dL, and 200 mg/dL for HDL, LDL, TG, and TC, respectively). The reference group for each lipid profile was classified differently depending on the abnormal state of the figure. The analysis between HDL and DSST (beta coefficient: 3.113) showed a positive relationship and the analysis between HDL and TG (beta coefficient: −1.837) presented a negative relationship; however, LDL and TC did not show statistical significance. A group classified as having high HDL was more likely to show a higher score for the cognitive test than a group with low HDL. Moreover, a group classified as having low TG was more likely to show a lower score for the cognitive test than a group with high TG.

A low DSST score represented a high likelihood of cognitive impairment from our methodology in logistic regression analysis. The DSST score showed no relationship with continuous values of the lipid profiles. For categorical values, HDL was only associated with a 0.701 times increased risk of cognitive impairment (95% CI = 0.523–0.938). A group classified as having high HDL indicated a decreased risk of cognitive impairment.

## 4. Discussion

We investigated the association between serum lipid parameters and cognitive performance (assessed using the DSST) of the civilian elderly population in the United States. We found that older adults with higher blood concentrations of HDL had good DSST performance. This association was robust regardless of age, sex, ethnicity, income, education, smoking, exercise, BMI, depressive feelings, diabetes, chest pain, hypertension, and statin, in both the multiple linear regression model and logistic regression analysis. Moreover, our findings suggest that the low TG group was more likely to show a lower score for the cognitive test compared to the high TG group. LDL and TC levels were not associated with the DSST. Our findings indicate that HDL may be protective against cognitive performance decline.

Consistent with our findings, some previous studies have suggested the beneficial role of HDL-C in cognitive function [11,21,22]. A cross-sectional study of 540 elderly individuals in central New York showed that those with higher levels of HDL (≥60 mg/dL) scored much better on the Mini-Mental State Examination (MMSE), working memory, and global composite than their counterparts. Specifically, the mean z-scores (±standard error [SE]) observed in subjects with HDL ≥ 60 mg/dL vs. those with HDL < 40 mg/dL were 0.195 (0.076) vs. 0.000 (0.000) *p* < 0.05, MMSE and 0.192 (0.081) vs. 0.000 (0.000); *p* < 0.05 for working memory [11]. A study in Western Australia reported that HDL delayed the onset of Alzheimer’s disease and dementia. The beneficial influence of HDL on verbal episodic memory has presented great relevance. HDL cholesterol was the most significant predictor of California Verbal Learning Test (CVLT)—short delay free recall (β = 0.251, SE = 0.776, *p* < 0.01) and Discriminability score (β = 0.261, SE = 1.559, *p* < 0.05), among other verbal learning and memory tests [21]. A similar result regarding the positive aspect of HDL was found in a cohort of nonagenarians in the Mugello study. Multiple linear regression analysis showed a significant relationship between HDL and MMSE scores (β = 0.174, *p* = 0.037) in male individuals [22]. In contrast, a Chinese longitudinal study based on 2291 participants aged >60 years found no significant association between HDL and cognitive decline during the follow-up period [23]. This discrepancy may be due to the differences in the normal expected range of HDL assessment and sample participants’ characteristics. Further studies are needed to identify the HDL-cognition link.

Unfortunately, we were unable to find a significant relationship between TC and LDL and DSST scores. Although our study found an association between TG and the DSST scores (as shown in Table 4), only the multivariate linear regression analysis for the categorical groups showed the findings considering the different results from Table 3. Recent general literature reports have summarized the inconsistent results of lipid cognition [24,25]. Specifically, a study of Chinese cohort of 709 nonagenarians and centenarians (mean age 93.8 years) found no association between the levels of serum lipid/lipoprotein and mean MMSE score [26]. A longitudinal cohort study from northern Manhattan showed that plasma lipid levels do not affect the changes in cognitive performance of memory, language, and visuospatial abilities in the elderly followed up for 7 years [27]. Furthermore, few animal studies related to lipid-lowering treatment regarding cognitive enhancement have revealed conflicting results [28,29]. A possible explanation for the different results of lipid cognition may be the characteristics of the study population, study design, different guidelines, and the disturbance of cognitive performance measurements. In addition, the lipid levels at the age of the starting point of the study did not fully cover the earlier life of lipid levels. Although the different effects of lipid profile on cognitive impairment may vary depending on several conditions, an important interpretation of our study result implies that lipid cognition research still needs more attention. Evaluating the levels of lipid profile could be an early vital sign of cognitive impairment.

Herein, we are concerned with the reason for the association of HDL concentrations with poor cognitive performance. The underlying mechanism is still unclear, although the connection of HDL to cerebral and cerebrovascular diseases may help in understanding their link [22]. HDL cholesterol enables the removal of excess cholesterol from cells and carries it to the liver [22]. This process protects the arteries and prevents atherosclerosis [22]. High levels of HDL cholesterol have been related to the volume of hippocampal and damaged Aβ fibrillization [30]. In addition, it may serve as a cardiovascular protectant against reversing cholesterol transport and improving the delivery of apolipoprotein E (ApoE) [31,32]. The protective role of HDL helps preserve the regions around the brain concerned with verbal memory [21]. The levels of HDL have been examined in relation to verbal memory deficits in middle-aged adults from the Whitehall II study [33]. Given that memory deficit in the brain region is highly associated with cognitive function, a lower baseline of HDL is relevant to the temporal regions of lower gray matter volumes [34]. The positive link between HDL cholesterol and the cardiovascular system is likely to achieve high scores on cognitive performance tests.

Our study had some limitations. We used the public NHANES dataset, which offered a fraction of cognitive variables. Therefore, it was difficult to obtain multiple screening tools for cognitive impairment measurements and identification of individual participant’s ApoE genotype, which is known to be a genetic risk factor for cognitive and cardiovascular diseases. In addition, the inclusion of several factors, such as levels of serum insulin, serum cortisol, plasma Aβ, and thyroid hormones, would have strengthened our results. Since this study was cross-sectional, it was relatively difficult to explain the causal relationship between cognitive decline and lipid profiles. Therefore, our study may be further enhanced by expanding future longitudinal studies examining time relations among multiple cognitive domains. A longitudinal analysis would be able to track an individual’s cognitive decline and easily determine the long-term clinical significance [21].

## 5. Conclusions

In conclusion, our study reported that higher HDL levels were positively associated with the DSST. Our study suggests that reduced HDL levels may be a factor associated with poor cognitive function. Modifiable lifestyle behavior changes and ingestion of a balanced healthy diet to maintain high HDL levels may be essential for better cognitive function in older adults.

## Figures and Tables

**Table 1:** Characteristics of the study population (*n* = 2215).

Variables	Mean (SD) or *n* (%)
Age (y)	68.96 ± 6.65
Sex	
Male	47.27 (%)
Female	52.73 (%)
Race/Ethnicity	
Mexican American	9.16 (%)
White	47.22 (%)
Black	23.34 (%)
Asian	8.26 (%)
Other Race	12.01 (%)
Income	
Low	24.24 (%)
High	75.76 (%)
Education	
Non High School Grad	23.79 (%)
High School Grad	23.02 (%)
College Grad or above	53.18 (%)
Smoking	
Non experience at all	12.33 (%)
Experienced at the past	36.30 (%)
Current Smoking	51.38 (%)
Exercise	
Non	40.27 (%)
Regularly	59.73 (%)
BMI (kg/m^2^)	
Underweight	1.58 (%)
Normal	26.95 (%)
Overweight	36.03 (%)
Obesity	37.02 (%)
Depressive feeling	
Yes	22.84 (%)
No	77.16 (%)
Diabetes	
Yes	4.56 (%)
No	95.44 (%)
Chestpain	
Yes	20.90 (%)
No	79.10 (%)
Hypertension	
Yes	35.25 (%)
No	64.75 (%)
Statin	
Yes	37.79 (%)
No	62.21 (%)

Income: low (total annual family income <$20,000) and high (total annual family income ≥$20,000).

**Table 2:** Comparison of the means (SD) of lipid profiles and cognitive test score.

	Mean	SD
Lipid profiles		
HDL	55.11	16.27
LDL	113.05	35.35
TG	120.89	70.9
Total Chol	194.61	42.63
Cognitive Test		
DSST	47.27	17.33

HDL: high density lipoprotein; LDL: low density lipoprotein; TG: triglyceride. Total Chol: total cholesterol. DSST: digit symbol substitution test.

**Table 3:** Pearson correlation structure of lipid profiles-DSST and among lipid profiles.

	DSST		
	No.	R	*p*-Value
HDL	2215	0.127	<0.0001
LDL	1078	0.107	0.0005
TG	1088	0.019	0.5372
Total Chol	2215	0.111	<0.0001
	Lipid Profiles		
	No.	R	*p*-value
HDL-LDL	1078	0.079	0.0039
HDL-TG	1088	−0.419	<0.0001
HDL-Total Chol	2215	0.288	<0.0001
LDL-TG	1078	0.089	0.0012
LDL-Total Chol	1078	0.925	<0.0001
TG-Total Chol	1088	0.111	<0.0001

**Table 4:** Multiple linear regression and logistic regression analysis of the association between lipid profiles and DSST.

DSST	Beta Cofficient (SE)	*p*-Value	DSST (Low/High)	Odds Ratio (95% Confidence Limits)	*p*-Value
HDL	0.036 (0.018)	0.0447		0.994 (0.986–1.002)	0.1506
LDL	0.018 (0.011)	0.1166		0.998 (0.993–1.004)	0.5631
TG	0.003 (0.005)	0.5689		1.000 (0.998–1.002)	0.9897
Total Chol	0.013 (0.007)	0.0514		0.997 (0.994–1.000)	0.0852
Categorization of Lipid Profiles					
${\mathrm{HDL}\;}^{a}$					
High	3.113 (0.647)	<0.0001		0.701 (0.523–0.938)	0.0167
Low					
${\mathrm{LDL}\;}^{b}$					
High					
Low	−0.837 (0.925)	0.3658		1.420 (0.946–2.131)	0.0911
${\mathrm{TG}\;}^{c}$					
High					
Low	−1.837 (0.920)	0.0461		1.194 (0.798–1.788)	0.3888
${\mathrm{Total}\;\mathrm{Chol}}^{\;d}$					
High					
Low	−0.468 (0.574)	0.4151		1.131 (0.875–1.462)	0.3457

${\mathrm{HDL}}^{\;a}$ High: >40, Low: ≤40; ${\mathrm{LDL}\;}^{b}$ High: >130, Low: ≤130; ${\mathrm{TG}}^{\;c\;}$ High: 150, Low: <150; ${\mathrm{Total}\;\mathrm{Chol}\;}^{d}$ High: ≥200, Low: <200, Model was adjusted for age, gender, ethnicity, income, education, smoking, exercise, BMI, depression, diabetes, chest pain, hypertension and statin.

## Data Availability

Data can be downloaded from the “NHANES” database (https://www.cdc.gov/nchs/nhanes/index.htm, accessed on 12 September 2021).
